# Army Medical Corps Examinations

**Published:** 1907-04

**Authors:** 


### ARMY MEDICAL CORPS EXAMINATIONS.

---

Preliminary examinations for appointment of Assistant Surgeons in the Army will be held on April 29th and July 29th, 1907, at points to be hereafter designated.

Permission to appear for examination can be obtained upon application to the Surgeon General, U. S. Army, Washington, D. C., from whom full information concerning the examination can be procured. The essential requirements to securing an invitation are that the applicant shall be a citizen of the United States, shall be between twenty-two and thirty years of age, a graduate of a medical school legally authorized to confer the degree of doctor of medicine, shall be of good moral character and habits, and shall have had at least one year's hospital training or its equivalent in practice. The examinations will be held concurrently throughout the country at points where boards can be convened. Due consideration will be given to the localities from which applications are received, in order to lessen the traveling expenses of applicants as much as possible.

In order to perfect all necessary arrangements for the examinations of April 29th, applications must be complete and in possession of the Surgeon General on or before April 1st. Early attention is therefore enjoined upon all intending applicants.

There are at present twenty-five vacancies in the Medical Corps of the Army.

